# Membrane Distillation and Other Membrane-Related Applications for Water Cleaning and Desalination

**DOI:** 10.3390/membranes16030081

**Published:** 2026-02-25

**Authors:** Simona Renda

**Affiliations:** Catalysis and Reactor Engineering Group (CREG), Aragon Institute of Engineering Research (I3A), Universidad de Zaragoza, 50018 Zaragoza, Spain; srenda@unizar.es

## 1. Introduction

The increasing demand for clean and safe water across the globe represents one of the most pressing challenges of modern society. Population growth, industrialization, agricultural activities, and climate change are placing severe stress on conventional freshwater resources, accelerating the need for advanced and sustainable water purification technologies. Against this background, membrane distillation (MD) has undergone a significant “research boom” over the past decade, evolving from a niche seawater desalination method into a pivotal technology in various complex industrial wastewater treatments.

## 2. Overview of Special Issue Contributions

Membrane distillation is a thermally driven membrane process that exploits a hydrophobic, microporous membrane to separate a warm feed stream from a cooler permeate side [1]. The resulting vapor pressure difference enables the selective transport of water vapor through the membrane’s pores while effectively retaining non-volatile species such as salts, organic compounds, and pathogens. Unlike pressure-driven membrane processes, MD operates at low hydraulic pressure and moderate temperatures, making it well suited for coupling with low-grade waste heat and renewable energy sources, including solar and geothermal energy [2].

A key advantage of membrane distillation is its ability to achieve a theoretically complete rejection of dissolved contaminants while maintaining stable performance, even at very high salinities [3]. This makes MD particularly attractive for challenging applications such as seawater desalination [4], hypersaline brine concentration [5], industrial wastewater treatment [6], and the removal of emerging contaminants [7] and microplastics [8]. In addition, MD systems’ modularity and operational flexibility support their potential deployment in decentralized and small-scale water treatment configurations [9].

However, despite its promising characteristics, progress in the application of MD has been hindered by a lack of specifically designed membranes, recurrent pore wetting, high energy consumption compared to reverse osmosis, and limited consensus on mathematical modeling for heat and mass transfer. For this reason, the technique is still under active development, with research efforts focused on addressing limitations related to membrane wetting, fouling, thermal efficiency, and long-term operational stability. Recent developments in membrane material design, surface functionalization, module configuration, and process integration has led to significant performance improvements, reinforcing MD’s position as an innovative and versatile technology within the broader membrane science landscape.

## 3. Conclusions

This Special Issue, “Membrane Distillation and Other Membrane-Related Applications for Water Cleaning and Desalination”, aims to capture recent advances in membrane distillation and related water purification technologies. It comprises one comprehensive review article, which critically examines the state of the art in MD, alongside nine original research papers, addressing critical knowledge gaps through a multidisciplinary collection of research focused on advanced materials, process modeling, and practical industrial implementation.

Together, these contributions provide a coherent overview of current research trends and highlight emerging directions that are expected to drive future developments in the field.

Despite these advancements, future research must prioritize the scaling up of these processes into continuous industrial operations and the development of multi-fiber modules to maximize effective surface area. Priority should be given to the design of nano-engineered or 3D-printed modules that allow for unprecedented control over temperature polarization and membrane architecture. Moreover, future efforts should integrate molecular-simulation-driven precision to target emerging contaminants such as PFAS and microplastics, ensuring that membrane technologies remain a sustainable and economically viable cornerstone of global water security.
